# Development of a Conceptual Framework for Severe Self-Neglect (SN) by Modifying the CREST Model for Self-Neglect

**DOI:** 10.3389/fmed.2021.654627

**Published:** 2021-05-17

**Authors:** Sabrina Pickens, Mary Daniel, Erick C. Jones, Felicia Jefferson

**Affiliations:** ^1^Department of Research, University of Texas Health Science Center at Houston, Jane and Robert Cizik School of Nursing, Houston, TX, United States; ^2^College of Engineering, Industrial, Manufacturing and Systems Engineering Department, University of Texas Arlington, Arlington, TX, United States; ^3^Biology Academic Department, Fort Valley State University, Fort Valley, GA, United States

**Keywords:** self-neglect, artificial intelligence, sleep, geriatrics, cognition

## Abstract

Self-neglect is an inability or refusal to meet one's own basic needs as accepted by societal norms and is the most common report received by state agencies charged with investigating abuse, neglect and exploitation of vulnerable adults. Self-neglect is often seen in addition to one or multiple conditions of frailty, mild to severe dementia, poor sleep and depression. While awareness of elder self-neglect as a public health condition and intervention has significantly risen in the past decade as evidenced by the increasing amount of literature available, research on self-neglect still lacks comprehensiveness and clarity since its inception to the medical literature in the late 1960s. With the burgeoning of the older adult population, commonness of self-neglect will most likely increase as the current incidence rate represents only the “tip of the iceberg” theory given that most cases are unreported. The COVID-19 pandemic has exacerbated the incidence of self-neglect in aged populations and the need for the use of intervention tools for aging adults and geriatric patients living alone, many of which may include in-home artificial intelligence systems. Despite this, little research has been conducted on aspects of self-neglect other than definition and identification. Substantial further study of this disorder's etiology, educating society on early detection, and conceivably preventing this syndrome altogether or at least halting progression and abating its severity is needed. The purpose of this research is to provide a definition of severe self-neglect, identify key concepts related to self-neglect, comprehensively describe this syndrome, present a conceptual framework and analyze the model for its usefulness, generalizability, parsimony, and testability.

## Introduction

Self-neglect is the inability or refusal to meet one's own basic needs as accepted by societal norms (1). It is a commonly seen by practitioners in association with geriatric conditions of frailty and characterized by losses in physical, psychological, and social domains. Self-neglect is the most common report received by Adult Protective Services (APS), state agencies charged with investigating abuse, neglect and exploitation of vulnerable adults (2–4). Some studies indicate that older adults who self-neglect have two and half times the mortality rate than adults who have never been reported to APS (5) and ~2 times the 1-year mortality rate of adults who do not self-neglect (6). Other studies indicate rates as high as a six-fold increase in 1-year mortality of self-neglecters with as high as a 15-fold increase of mortality for individuals with self-neglect compared to those without self-neglect (7). With the burgeoning of the older adult population, the commonness of self-neglect will most likely increase as the current incidence rate represents only the “tip of the iceberg” theory given that most cases are unreported (8).

While awareness of elder self-neglect as a public health condition meriting investigation and intervention has significantly risen in the past decade as evidenced by the increasing amount of literature available, research on self-neglect still lacks comprehensiveness and clarity since its inception to the medical literature in the late 1960s. The literature found by these authors focuses almost exclusively on identifying and defining self-neglect, which has been a critical undertaking given the dearth of literature prior to the last decade. A number of studies report the complex nature of self-neglect, yet the lack of a standard definition or standard assessment tool makes it difficult to study and to mutually recognize its occurrence among health care professionals (1, 9–17). To date, the National Association of Adult Protective Service Administrators (NAPSA) provides the most comprehensive definition of self-neglect as:

an adult's inability, due to physical or mental impairment or diminished capacity, to perform essential self-care tasks including: (a) obtaining essential food, clothing, shelter, and medical care; (b) obtaining goods and services necessary to maintain physical health, mental health, emotional well-being, and general safety; and (c) managing one's own financial affairs excluding an individual's lifestyle choice [(18), p. 33].

Another comprehensive definition provided by the National Centers of Elder Abuse, which excludes conscious, voluntary decisions by mentally competent adults is:

the behavior of an elderly person that threatens his/her own health and safety. Self-neglect generally manifests itself in an older person as a refusal or failure to provide himself/herself with adequate food, water, clothing, shelter, personal hygiene, medication (when indicated), and safety precautions (19).

On the other hand, the common definition in the literature states self-neglect is the inability to meet one's own basic needs or behaviors of an individual that threatens his or her self-care (8, 20–23). These definitions are either lengthy and/or do not reflect the seriousness of elder self-neglect if left unrecognized and untreated.

These authors study self-neglect, see self-neglect in an emergency responder capacity, and/or have medically treated this population. Since little research has been conducted on aspects of self-neglect other than definition and identification, this disorder lends credibility to a life-long, academic pursuit in studying its etiology, educating society on early detection, and conceivably preventing this syndrome altogether or at least halting progression and abating its severity.

Walker and Avant (24) illustrate the processes of concept synthesis, derivation, and analyses. Application of concept synthesis is to encompass the varying definitions of self-neglect to develop a standard definition and conceptual framework. In conducting concept synthesis, a mixed method approach will be undertaken. This method includes a review of the literature and qualitative synthesis based on the authors' own observations as well as collaboration with experts in the field.

The literature review included searches in PUBMED, SCOPUS, MEDLINE, CINNAHL, Administration on Aging, NAPSA, and the National Center on Elder abuse using the following key terms: self-neglect, older adults, senile breakdown syndrome, social breakdown, Diogenes' syndrome, elder abuse, elder mistreatment, neglect, elder neglect, executive dysfunction, and impaired cognition. Inclusion criteria for article selection are those written in English and persons 65 years of age and older who self-neglect. Exclusion criteria include persons <65 years of age, focusing within specific ethnic groups or nationalities, and other forms of elder abuse such as caregiver neglect, financial exploitation, and physical abuse without mention of self-neglect. The purpose of this paper is to provide a definition of severe self-neglect, identify key concepts related to self-neglect, comprehensively describe this syndrome, present a conceptual framework and analyze the model for its usefulness, generalizability, parsimony, and testability.

### Key Concepts

Self-neglect occurs along a continuum ranging from mild to severe in nature (17). For the purpose of this conceptual framework, severe self-neglect will be used. Severe self-neglect is defined as an unawareness to the hazardous and progressive decline in personal, social, physical, mental, and/or environmental domains leading to the inability to maintain culture and community standards of acceptable living that threatens one's own safety, health, and quality of life. The purpose of this definition is to define a phenomenon that is complex and progressive yet clearly highlights the lack of awareness to one's hazardous state of health (17).

**Figure 1:**
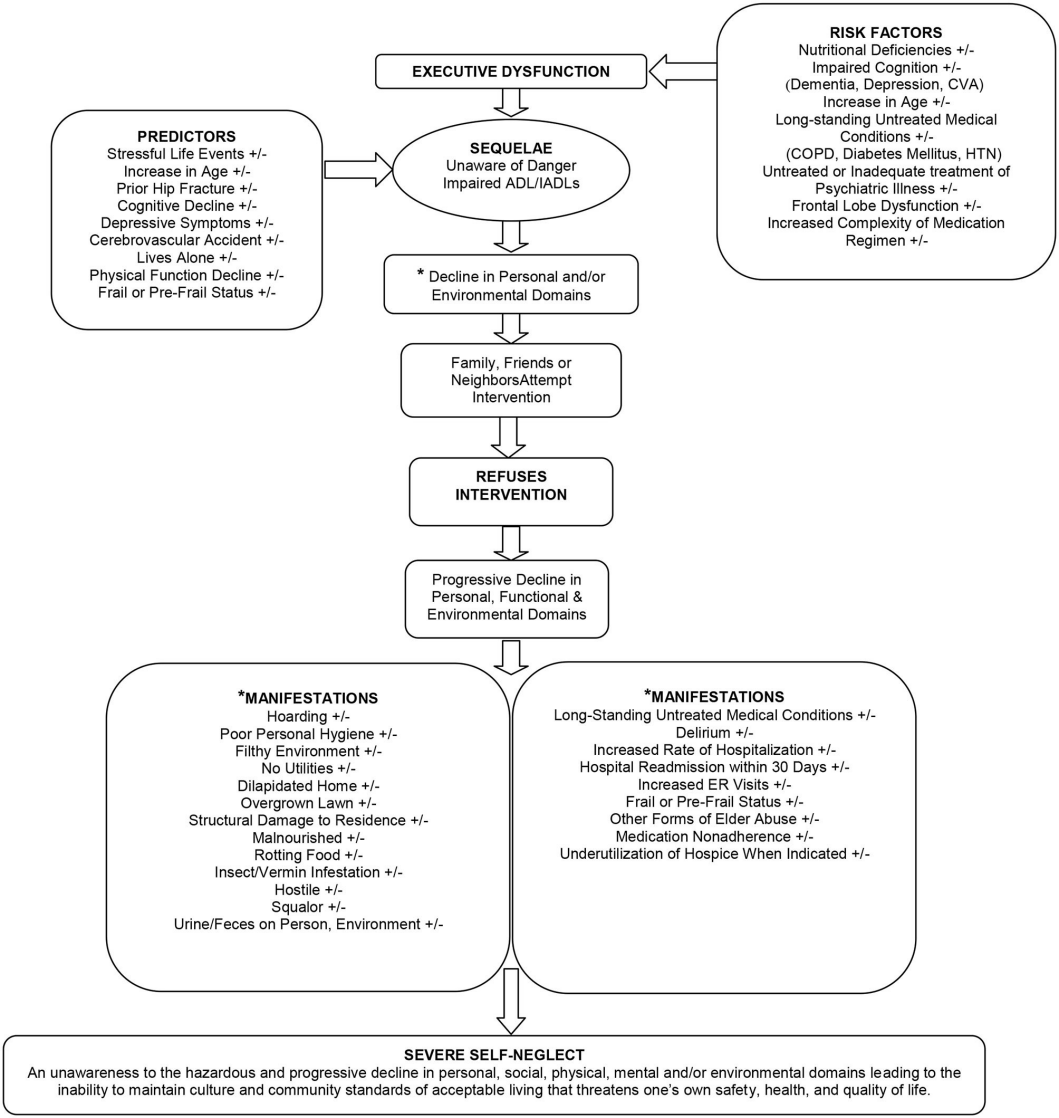
Conceptual framework for the etiology of severe self-neglect by modification of the CREST model of self-neglect.

The conceptualization of severe self-neglect is that it develops in the presence of known predictors or when one develops executive dysfunction as defined below in this paper. When either of these two factors is present, older adults develop functional disabilities, which may lead to a decline in their personal and/or environmental domains. Informal caregivers may attempt some form of intervention to aid self-neglecters, yet the self-neglecters refuse to accept help. When self-neglecters refuse nursing, medical, or social intervention, it is hypothesized that a progressive decline in their personal, functional, environmental, and social domains ensues. This decline manifests as poor personal hygiene, filthy environments, hoarding unnecessary items, malnourishment, rotting or spoiled food, or delirium, to name a few. What remains to be tested is the absolute presence, absence, or combination of the manifestations seen in self-neglecters (see Figure 1 for manifestations).

The etiology of severe self-neglect has not been clearly identified although known predictors resulting in self-neglect have been identified. Predictors are defined as symptoms or indicators present at the time of disease onset. Only five research articles were found to report physical predicting factors for self-neglect. Stressful life events, older age, a prior history of a hip fracture or cerebrovascular accident, objective physical function decline using physical performance testing, self-reported physical function decline using the Katz Index or Rosow-Breslau Health Scale, frail or pre-frail status on the Fried Frail Phenotype test, male gender, low income, cognitive decline, and depressive symptoms have all been identified as predictors to self-neglect (10, 21, 25–27).

#### Executive Dysfunction

Executive dysfunction (ED) is defined as the inability to complete complex tasks such as managing financial transactions or preparing meals, actions that require high cognitive abilities (28–30). Increasing complexity of the medication regimen has proven to be a risk factor contributing to ED and self-neglect with higher levels of regimen complexity resulting in lower levels of medication adherence (31). Medication non-adherence is a manifestation of self-neglect associated with increasing complexity of the medication regimen as well as lower physical functioning in self-neglecters (32). These results highlight the cyclical nature of self-neglect and medication in such that ED and comorbidities dictate a need for more medication which increases the risk of non-adherence which can exacerbate the ED and comorbidities. Moreover, executive dysfunction is the inability to initiate or halt actions, plan future events in the presence of novel tasks, inhibit inappropriate behaviors or alternate plans quickly when events interfere with an individual's usual routine of care (33). Several medical conditions such as major depression, diabetes mellitus, memory disorders, psychiatric illnesses, and frontal lobe dysfunction all have been associated with the development of ED (28). Brain atrophy associated with aging can also contribute to the onset of ED (29, 33). Executive dysfunction can be present even with a normal Mini-Mental State Examination (MMSE) score, as well as in highly educated individuals (29, 34).

Refusal is defined as the self-neglecter being unwilling to accept nursing, social, or medical interventions at the persuasion of family, friends, or neighbors. This refusal of care is in the presence of functional, personal, mental, and/or environmental decline. Refusal is a cardinal feature of self-neglecters and is often attributed to a life-style choice or eccentric living (14, 35). For the purpose of this framework, unawareness to dangerous living conditions indicates that eccentric life-style choices and behaviors are not optional and are self-neglect characteristics.

#### Lack of Standard Definition Decreases the Identification of Self-Neglect

Several authors have identified the need to define and conceptualize this problem in order to conduct more, rigorous research (1, 9, 11–17, 43). There is consensus among researchers that with a standard definition, comparison of study results among disciplines is possible. As of 2012, only 39 states and the District of Columbia include self-neglect in their state elder abuse statutes despite self-neglect being the most common allegation reported to APS agencies across the United States (44). Consequently, true incidence and prevalence rates are undetermined due to varying or lack of definitions which exist for self-neglect among states and researchers (see Table 1).

**Table 1:** Lack of standard definition decreases identification of Self-Neglect (SN).

**References**	**Purpose of study**	**Population (*N*)**	**Intervention**	**Outcomes**	**Authors' conclusion**	**Our conclusion**
Adams and Johnson (9)	SN is present in the medical literature and little attention to the nursing literature	Hospital and community nurses	Interviewed nurses to see if they could identify SN	All nurses were able to identify gross SN	Poor nutrition was a common feature identified with gross SN	Agree, and it has been identified in the literature (i.e., malnutrition, nutritional deficiencies)
Bozinovski (10)	Develop conceptual framework for SN	Qualitative interview of SNers and APS caseworkers (*N* = 70)	Qualitative interviews	Framework based on self-continuity of preserving/protecting self and maintaining customary control	Using the term SN is a misnomer rather older persons labeled as SN are engaged in a process of struggling to maintain coherency of self	Agree with some aspects of this; however, if someone is demented and lives alone with impaired ADL/IADLs it is doubtful they are attempting to maintain customary control or preservation
Dick (16)	Commentary	N/A	N/A	N/A	Need to develop a conceptual and operational definition for SN and diogenes syndrome to formalize the language, synthesize knowledge and reduce the labels applied to this population	We agree
Gibbons (15)	Propose SN as a NANDA diagnosis	N/A	Literature review	SN is either intentional or non-intentional; non-intentional is the failure to engage in self-care actions necessary for health and well-being as a result in deficits in cognition and other mental, physical, material, or social resources needed to participate in self-care	SN as a NANDA diagnosis is either intentional SN or non-intentional SN; Need a standard definition	Agree, but it doesn't describe the unawareness of the situation
Gunstone (13)	Explores the perceptions and experiences of community mental health workers who assess and manage the risk of SN and severe SN in persons with serious health problems	7 community mental health nurses	Semi-structured interviews	Nurses working in a number of areas where there is a distinct lack of clarity- “The Gray Areas” of which the most important were tolerance of workers to situations of SN/Severe SN, policies, procedures, legislation, and definitions	Need to balance safety needs of clients against their need to be treated as autonomous is a major dilemma with nurses; SN lacks a clear definition	We agree
Lauder (36)	Explore the medical constructs of SN	N/A	Literature review		SN is a symptomatic disorder of a fragmented phenomenon; recognizes unique and personal experience of each SN case vs. a universal definition; SN is a concrete human experience which must be understood within a particular historical context within its own cultures and values and interpersonal practices	Agree with parts but do not agree with the patients living in human and animal feces thinking it's ‘okay’. Clearly there's a disconnect
Lauder (1)	Explore the utility of self-care theory in understanding SN		Using SC theory to understand SN	Household squalor, poor diet, failure to look after one's health, poor personal hygiene, mental and physical health problems, inability to sustain and develop interpersonal relationships, homes dirty, littered, and disrepair	SC theory can explain some aspects of SN which may be due to our inability to explain human behavior leading to SN; it may be due to the lack of self-care theories	Agree since SN is a complex phenomenon
Lauder et al. (14)	Provide an overview of SN and a framework for managing this problem	N/A	Lit review	Severe household squalor, major decline in personal hygiene, housing disrepair, poor personal hygiene, household untidiness	SN is a violation of acceptable social norms; competing definitions	We agree that there are so many varying definitions leading to the confusion of diagnosing and treating SNers
Orem (12)	Letter to the Editor based on Lauder's theory of SN in the SC theory	N/A	N/A	N/A	SN needs conceptualization to determine validity and reliability of the formulated and expressed concepts as it's used in health care practices. There are no formal and expressed concepts of SN. We need a detailed description of a range of instances of SN to reveal a number of clear-cut cases with evidence of essential elements and relationships among them	We agree

Lauder applied self-neglect to Orem's Self-Care Theory; however, this theory does not encompass all features of self-neglect due to its complexity (1). In 2001, Dorothy Orem argued that self-neglect needs to first be conceptualized prior to utilizing this term in the health care arena (12). As it is outlined in the literature, self-neglect occurs along a continuum; however, the focus of this conceptual framework is on the extreme form along the continuum (1, 12, 17).

#### Executive Dysfunction Increases the Risk of Self-Neglect

Impairments in executive function contribute to institutionalization due to patient morbidity and subsequent caregiver burden (29, 30). Research demonstrates that executive function is significantly and independently associated with functional status in older adults (29, 39, 45). An independent association has been discovered between executive function decline and an increased risk of self-neglect; however, the study showed that global cognitive function, MMSE, and episodic memory are not independently associated with a greater risk of self-neglect (45). When individuals develop ED, their decisional making abilities are questionable (39). In assessing decision making autonomy, health care providers must address not only the patient's ability to make a decision, but also the ability to carry out that decision (28). In other words, the elder must articulate then demonstrate their decision. For example, in a study of elder self-neglecters, elders reported taking their medication as prescribed yet upon a total pill count, there were discrepancies between what the elder reported, the number of pills prescribed, and the number of pills left in the medication bottle (46).

Understanding, intentionality, and voluntariness are the three components of decisional making capacity (47). Individuals with ED lack the ability to make voluntary decisions and carry out these decisions. These two important components of decisional making autonomy are often not detected with traditional mental status examinations (39). Without detection, adverse health consequences occur in individuals thought capable of making informed decisions regarding their medical regimens (28).

Pickens et al. (38) conducted a cross sectional pilot study of 100 self-neglectors aged 65 and older and 100 community matched controls, which included administering the KELS and the MMSE (see Table 2). Analyses revealed self-neglecters were significantly more likely to fail the KELS compared to the control group even after stratifying by normal MMSE scores (38). These results may be due to impaired executive function not identified with MMSE testing. Although no prior research directly links ED to a lack of capacity, individuals who lack capacity also have ED (39). This linkage needs to be tested with future research.

**Table 2:** Executive dysfunction may increase the development of self-neglect.

**References**	**Purpose of study**	**Population (*N*)**	**Intervention**	**Outcomes**	**Authors' conclusion**	**Our conclusion**
Dyer et al. (23)	Characterize a group of self-neglectors 65 years and older	Older adults aged 65 years and older with validated SN referred to a medical team by APS (*n* = 538)	Cross sectional chart review	Average patient age was 75.6, 70% were female, 460 were 65 years of age and older, 50% had abnormal MMSE scores, 15% had abnormal GDS scores, 76.3% had abnormal PPT scores, 95% had moderate to poor social support based on the DUKE Social support index, multiple co-morbidities were noted yet more than 46% were on no meds	Underlying medical disorders leads to executive dysfunction resulting in impairments in IADLs. When there is a lack of social support services in this group, self-neglect ensues	CREST model of self-neglect needs to be adapted
Kohlman-Thomson (37)	N/A	N/A	N/A	N/A	Booklet with instructions on how to administer and score the KELS. In the introduction, the author describes its utility and how in some states it's used in the courts for determination of commitment and gravely disabled cases	
Pickens et al. (38)	Compare KELS scores between substantiated SN and matched community controls	65 years of age and older with substantiated SN by APS (*N* = 100)	CGA including the KELS	SN significantly more likely to fail the KELS compared to matched controls. When stratified by MMSE, SN with intact cognitive function still significantly more likely to fail the KELS	KELS provides clinicians with an objective measure of an individual's capacity and performance with everyday life-supporting tasks and thus provides information that can help NPs identify elders at risk for SN	We agree
Royall et al. (29)	To assess the contribution of executive control function to functional status	Non-institutionalized septuagenarians (*N* = 547)	MMSE, EXIT25, and functional status measurements conducted	The effect of the EXIT 25 on change in IADl was stronger than those of age, baseline IADLs, comorbid disease and level of care	ECF is a significant and independent correlate of functional status in normal aging	
Royall et al. (30)	To assess the contribution of changes in executive control function and memory to changes in functional status	Non-institutionalized septuagenarians (*N* = 547)	CVLT, EXIT25, functional status measurements conducted	EXIT25's effect on the rate of change in IADLs was stronger than those of age, baseline IADLs, comorbid disease or level of care	ECF is a strong, significant and independent correlate of functional status in normal aging. In contrast, memory decline has no independent association with rate of change in functional status	
Schillerstrom et al. (39)	A review of the impact of medical illness on ED and discuss practical diagnostic instruments and treatment strategies	N/A	N/A	N/A	Patients with ED are more likely to resist care and less compliant with medications. ED makes a significant contribution to impaired IADLs and longitudinal rates of change in ADL performance. Medical patients should be screened for ED. CLOX test can help detect ED	Agree
Workman et al. (28)	N/A	N/A	N/A	N/A	Impaired executive function affects intentionality and voluntariness. Evaluation of autonomous decision-making capacity is an ongoing process that requires integration of data from multiple sources and detailed questioning by an IDT	
Wecker et al. (33)	Determine if age is related to a decline in executive function	Individuals 20–79 years old (*N* = 112)	California trail making and stroop test administered	After controlling for the component skills, age had a significant effect on executive requirement (speed) but didn't have an effect on switching	Study confirms importance of partialling out components in the assessment of multidimension tasks; emphasizes specificity over generalizability when examining impact of age on cognition	

#### Known Predictors Increase Risk of Self-Neglect

Currently, there are no prospective, longitudinal studies as to the onset of self-neglect, but there are longitudinal studies regarding identification and related aspects of self-neglect. The literature describing this phenomenon is based on case studies, retrospective reviews of social services and Adult Protective Service reports, retrospective chart reviews, qualitative studies, and analysis of the Established Populations for Epidemiologic Studies of the Elderly (EPESE) cohort database. One exception is the landmark study by MacMillan and Shaw (35) that introduced self-neglect to the medical field. These physicians evaluated and treated 72 older adults with self-neglect and followed them over 4 years.

Of considerable note, the Chicago Health and Aging Project (CHAP) was a longitudinal study of older adults conducted in 3-year cycles beginning in 1993 which grew from 6,158 community-dwelling participants living in certain Chicago neighborhoods in 1993 to 9,056 participants in 2005. As of 2005, the total number of individuals found to have been reported to social services in that cohort was 1,820 (48). A plethora of data was collected, including data regarding individuals who were identified as possible self-neglecters and the traits accompanying them, often separated into mild, moderate, or severe cases of self-neglect (see Table 3). As of 2010, there were 4,627 individuals still participating in the study (6, 7, 25, 26, 48–52).

**Table 3:** Known predictors increases self-neglect.

**References**	**Purpose of study**	**Population (*N*)**	**Intervention**	**Outcomes**	**Authors' conclusion**	**Our conclusion**
Abrams et al. (21)	Assess the contribution of depressive symptoms and cognitive impairment to the prediction of SN in elders residing in the community	Data analysis of the EPESE data base (*N* = 2,812)	N/A	Risk factors to developing SN are males, older age, low income, living alone, history of hip fracture or stroke, cognitive impairment and depressive symptoms	Elders residing in the community who experience depressive symptoms or impaired cognition may be at risk for SN	We agree
Bozinovski (10)	Develop conceptual framework for SN	Qualitative interview of SNers and APS caseworkers (*N* = 70)	Qualitative interviews	Framework based on self-continuity of preserving/protecting self and maintaining customary control; there deviant behavior pushes families and friends away when help is suggested	Using the term SN is a misnomer. Rather, older persons labeled as SN are engaged in a process of struggling to maintain coherency of self	Agree with some aspects of this; however, if someone is demented, lives alone with impaired ADL/IADLs, it is doubtful they are attempting to maintain customary control or preservation
Dong et al. (25)	Assess the contribution of measurable physical function decline with the prevalence of SN	1,068 of the 5,570 participants of CHAP from 1993 to 2005 who were reported to APS for suspected SN	Physical performance test, Katz ADL scale, Nagi scale, and Rosow-Breslau scale	For every 1-point decline in the physical performance test and declines in the Katz ADL or Rosow-Breslau scales were associated with an increased risk of SN	Increased physical impairment is independently associated with increased risk of SN	We agree
Dong et al. (26)	Assess the contribution of physical and mental function decline with the prevalence of SN stratified by gender and by SN factor; lower health status increased risk of SN	4,627 older adults from CHAP; 1,645 men and 2,982 women	Katz ADL scale, MMSE, and health status	Risk of SN increased as health status decreased; for each impairment on the Katz ADL scale, risk of SN increased for women in the factors of overall SN, hoarding, unsanitary conditions, and personal hygiene and for men in personal hygiene; for each lower point on the MMSE, SN increased in the factors of overall self-neglect, hoarding, house in need of repair, and unsanitary conditions for both genders	As levels of physical function, health status, and cognitive dysfunction decline, risk increases for SN and personal or environmental hazards, which are prevalent in an urban, community-dwelling aging population	Agree, but we have found these hazards to be prevalent in community-dwelling aging populations regardless of urban, suburban, or rural classifications
Lee et al. (27)	Assess the contribution of frailty status to the prediction of SN	Older adults with APS-verified SN, *N* = 37	Fried Frailty Phenotype assessment	3% of SNers were robust, 62% were pre-frail, and 35% were frail indicating that frail or pre-frail status can predict SN; individuals who are pre-frail are twice as likely to become frail	Current interventions are wise to target pre-frail older adults to delay progression from pre-frail to frail	We agree

#### Self-Neglect Within Communities and Groups

The population Study of Chinese Elderly in Chicago (PINE Study), conducted from 2011 to 2013, focused on self-neglecting, community-dwelling U.S. Chinese older adults in the greater Chicago area. Within this population, the unique manifestation of suicidality and unique confounding factors of illiteracy and language barriers were discovered (53). However, based on current research available, these authors are presently unable to determine if the discoveries of the PINE study can be generalized to all populations of self-neglecters or if they are confined to specific ethnicities, so suicidality, illiteracy, and language barriers are not included within this framework.

A study conducted on elder abuse and neglect among veterans among greater Los Angeles examined the prevalance, type, and intervention outcomes of elder abuse/neglect among a veteran population. A review of medical records of 575 veterans who had received services from the Veteran's Affairs Geriatric Outpatient Clinic in Los Angeles during a 3-year period found 31 veterans (5.4%) who had an elder abuse report filed on their behalf. Prevalance of elder abuse/neglect was higher among older (80+) and Caucasian and African American veterans. Eight of 31 victims suffered from more than one type of elder abuse including self-neglect (54). A close look at this study reveals that it lacked more information about self-neglect of veterans possibly resulting from PTSD (Post Traumatic Stress Disorder) and this can be further explored to prevent future cases on self-neglect in veterans.

#### Depression and Cognitive

Depressive symptoms and cognitive impairment in older adults were used to predict self-neglect by analyzing data from the New Haven EPESE cohort (21). Individuals were 65 years of age and older residing in the community. Male gender, older age, low income, depressive symptoms, impaired cognition, and a prior hip fracture or cerebrovascular accident were found to predict self-neglect. These outcomes were confirmed with Connecticut's investigations between 1982 and 1991. In Bozinovski's qualitative study interviewing self-neglecters and caseworkers, the development of self-neglect was attributed to a major, life-changing event such as death of a loved one or a decline in personal health (10).

#### Decline in Physical Condition

Measured declines in physical and cognitive function can predict the prevalence of self-neglect. A study by Dong et al. (26) as part of the CHAP population survey utilized the MMSE and the 6-item Katz Activities of Daily Living (ADL) scale to assess cognitive function and measure limitations for performing basic self-care, respectively. Results showed that for each impairment on the Katz ADL scale, the prevalence of overall self-neglect, hoarding, and unsanitary conditions increased significantly in women, and inadequate personal hygiene increased for men and women, indicating that a measurable decline in physical function can contribute to self-neglect. For each lower point on the MMSE, there was a substantial increase in overall self-neglect, hoarding, house in need of repair, and unsanitary conditions for both men and women, which corroborates the findings of impaired cognition contributing to self-neglect as seen in the New Haven EPESE cohort. Additionally, the study statistically proved that lower health status correlates with increased prevalence of self-neglect. A longitudinal CHAP-related study by Dong et al. (25) focused on assessing physical function by objectively measuring decline in physical performance testing, and measuring self-reported declines in the Katz, Nagi, and Rosow-Breslau scales. The results concluded that there was an increased risk of self-neglect with every 1-point decline in the physical performance test as well as with each decline in the Katz and Rosow-Breslau scales. There was no association between increased risk of self-neglect and a decrease in the Nagi scale.

#### Frailty

A study conducted by Lee et al. (27) used the Fried Frailty Phenotype (FFP) in assessing the abilities of self-neglecting older adults as it had been widely presumed that frailty contributes to self-neglect. Characteristics assessed with the FFP included unintended weight loss, self-reported fatigue, low physical activity, decreased grip strength, and slow walking speed. If none of the characteristics applied, the individual was considered robust; if one or two characteristics were present, the individual was classified as pre-frail; an individual with three or more applicable characteristics was considered frail. As expected, more self-neglecters were frail as opposed to robust; however, the majority of self-neglecting adults in the study were in the pre-frail status (27). Therefore, a predicting factor for severe self-neglect is frail or pre-frail status on the FFP (see Table 3).

#### Personality Traits

One zone of predictive factors, which was found to be statistically insignificant, is personality traits. Another part of the CHAP population survey, a study was conducted to determine whether the personality traits of neuroticism, extraversion, information processing, and rigidity were associated with the development of elder self-neglect. Though initial results indicated a positive association, it was determined that potential confounding factors were responsible for the association. Once the potential confounders were accounted for, there was no longer a statistically significant association between personality traits and self-neglect (48). Therefore, we did not include the results in our conceptual framework.

#### Sleep

Sleep quality and self-neglect in aged adults has been minimally explored (55), evaluated the association of elder abuse and poor sleep in a rural older Malaysian population. Researchers found self-neglect was significantly associated with poor sleep and recommended the creation of interventions or treatment modalities that focus on improving sleep quality among elder self-neglect and abuse populations. Additional research has looked at the impact of poor sleep quality in associated conditions (56–58). However, further research is needed in this area to assess the association of self-neglect with poor sleep quality.

Based on the authors' experience, self-neglect occurs in both genders regardless of income. Therefore, for the purpose of this conceptual framework, known predictors will be limited to stressful life events, older age, cognitive impairment, depressive symptoms, a history of either a stroke or hip fracture, physical function decline, or frail or pre-frail status. All of these disorders can lead to functional decline (25–27, 59–63). While income is not considered a predictor of the onset of severe self-neglect, a lack of income has been proven to contribute to a lack of ability to pay for medications and other medical treatments, which compounds the previously discussed risk factor of medication non-adherence (64).

#### Client Refusal Increases Risk of Self-Neglect

Commonly, self-neglecters come to the attention of APS or medical professionals when informal caregivers can no longer tolerate the self-neglecter's state of living (65). These individuals refuse medical attention, home cleaning, or removal from their home. Typically, they are not bothered by their personal and environmental domains despite the presence of urine and feces on floorboards, matted hair, overgrown nails, clutter, overgrown lawns, broken windows, or rotting food in their cupboards or refrigerators (1, 2, 11, 13–15, 40–42, 66–68).

In Texas, APS has the option of emergency removals, but this option may not be available in other states or countries (69). Commonly, involuntary removal is the only means for providing any intervention (69). Once hospitalized, treatment is initiated, yet upon discharge, self-neglecters refuse outpatient services (70, 71). Americans are very independent, so the least restrictive alternative to intervention is imperative. However, when self-neglecters lack the capacity for self-care and protection, they are no longer safe to live alone (17).

Lee et al. (72) successfully conducted the first known clinical intervention in a group of APS-substantiated self-neglecters, proving that not every self-neglecter will refuse intervention. Of the 94 possible referrals, there were 59 individuals who agreed to participate and 35 who completed the two-phase trial. However, severity of the self-neglect does not appear to have been taken into account. Therefore, client refusal of care is still included in the conceptual framework as a defining characteristic of severe self-neglect. This study should be considered in future research regarding mild to moderate self-neglect as it proves positive treatment outcomes for those who agree to the proposed intervention (see Table 4).

**Table 4:** Refusing intervention increases self-neglect.

**References**	**Purpose of study**	**Population (*N*)**	**Intervention**	**Outcomes**	**Authors' conclusion**	**Our conclusion**
Bozinovski (10)	Develop conceptual framework for SN	Qualitative interview of SNers and APS caseworkers (*N* = 70)	Qualitative interviews	Framework based on self-continuity of preserving/protecting self and maintaining customary control; there deviant behavior pushes families and friends away when help is suggested	Using the term SN is a misnomer rather older persons labeled as SN are engaged in a process of struggling to maintain coherency of self	Agree with some aspects of this however if someone is demented, lives alone with impaired ADL/IADLs I doubt they are attempting to maintain customary control or preservation
Clark et al. (40)	describe gross neglect in old age	Elderly patients admitted to a hospital in acute illness and extreme SN (*N* = 30)	Stabilization of medical problems	All had dirty, untidy homes, filthy personal appearance, 1/3 persistently refused help; acute presentation with falls was common, deficiencies in iron, folate, B12, vitamin C, calcium and vitamin D; high mortality rate (46%); personality characteristics aloof, suspicious, emotionally labile, aggressive, reality disoriented	These features might be called diogenes syndrome	I agree
Cooney and Hamid (41)	N/A	N/A	N/A	N/A	Main obstacle in helping SNers is their reluctance to seek help and resistance to medical intervention when offered. Need to gradually develop rapport and then encourage them to accept services	Agree with both suggestions except in underlying psychosis or untreated psychiatric disorders
Lauder et al. (14)	Provide an overview of SN and a framework for managing this problem	N/A	Lit Review	Severe household squalor, major decline in personal hygiene, housing disrepair, poor personal hygiene, household untidiness, service refusal	SN is a violation of acceptable social norms; competing definitions	I agree that there are so many varying definitions leading to the confusion of diagnosing and treating SNers
Reifler (42)	Editorial on diogenes syndrome	N/A	N/A	N/A	Diogenes syndrome also known as senile squalor, senile SN or social breakdown is characterized by social withdrawal, self-induced abysmal living and lack of concern about receiving assistance. There is evidence that these patients could be treated such as depression in conjunction with severe medical illness	Agree

While refusal of interventions increases the risk of self-neglect, self-neglect increases the risk of emergency and end of life healthcare utilization. Self-neglecters are more likely to need hospice services than elders who do not self-neglect, with severe self-neglecters experiencing the greatest increase in risk. There is also a decreased length of time spent on hospice care and time between admission into hospice care and death for individuals who self-neglect (7). Emergency department usage rates for elders who self-neglect were found to be significantly higher, roughly triple, that of those who do not self-neglect, with a gradient increase in severity of self-neglect and emergency department use (51). Additionally, it was determined that elders who self-neglect have an increased rate of hospital readmission within 30 days after being discharged, again roughly triple the rate of those who do not self-neglect, with a gradient increase in severity of self-neglect and hospital readmission (50).

### Recent Measurement Tools and Interventions

The Kohlman Evaluation of Living (KELS) assesses both basic and instrumental activities of daily living (37). The KELS relies on the individual's performance and self-report as well as observation by the administrator. In some states, the KELS is used in guardianship hearings to determine if someone can safely live by him/herself in the community based on their KELS' scores.

The Self-Neglect Severity Scale (SSS), proposed in 2006 by the Consortium for Research in Elder Self-neglect of Texas (CREST), laid the foundation for self-neglect severity assessment. It utilized questions clustered into the sections of personal hygiene; assessment of cognitive, health, and safety issues; and environmental assessment to determine the severity of self-neglect exhibited by an individual (2). Upon testing, the SSS was found to have sensitivity and specificity below the conventional acceptable range, thus necessitating an improved scale for a gold-standard screening tool (73). As of yet, no refinements to the SSS have been published; however, there have been several other assessment tools devised in the past decade to identify self-neglect and/or assess its severity.

Of note are the SN-37, the Elder Self-Neglect Assessment, the Abrams Geriatric Self-Neglect Scale, the IMSelf-Neglect questionnaire, and the Chicago Health and Aging Self-Neglect Scale. Many of these scales expand upon the foundation laid by the SSS (6, 74–77). The SN-37 is an instrument comprised of 37 items separated into 5 factors that contribute to self-neglect: environment, social networks, emotional and behavioral liability, health avoidance, and self-determinism. A provider based on the yes or no answers indicating the presence or absence of each item can suggest appropriate interventions. The SN-37 tool is comprehensive while remaining brief enough to avoid overwhelming the individual or the professional administering and interpreting the assessment (74). The primary limitation noted by these authors as SN-37 relates to this conceptual framework is that there did not appear to be a classification of the scoring system to define where a self-neglecter sits on the continuum from mild to severe self-neglect except that a higher score means increased severity. The purpose of SN-37 as understood by these authors was to focus more on interventions based on responses and not on classification; however, this conceptual framework attempts to define self-neglect at the severe end of the continuum and thus requires classification.

The Elder Self-Neglect Assessment (ESNA) is a psychometrically sound self-neglect assessment tool which has a long form of 62 items and a short form of 25 items. In both forms, items are divided into either behavioral characteristics or environmental factors. Using the Rasch item response theory and traditional validation approaches, it was determined that behavioral characteristics were more frequently associated with low to moderate severity of self-neglect while environmental factors were more frequently associated with moderate to high severity. The assessment is organized so that the questions are relatively in order from least to most severe (75). Like SN-37, the primary limitation noted by these authors as ESNA relates to this conceptual framework is the lack of classification of what scores constitute mild, moderate, or severe self-neglect.

The Abrams Geriatric Self-Neglect Scale (AGSS) is a 6-item assessment, but each item is its own factor with a handful of supporting questions to determine the score. The items are prescription medications, personal care, nutrition, environment/housing, financial stewardship, and socialization. The supporting questions help determine a score for each item of 0–4 for a total score range of 0–24, 0 meaning a complete absence of self-neglect and 24 meaning the most severe self-neglect (76). Given that the AGSS also does not classify which range of scores indicate mild, moderate, or severe self-neglect, the same limitation applies as it relates to this conceptual framework.

The Istanbul Medical School Elder Self-Neglect (IMSelf-neglect) questionnaire is an 11-item screening tool developed to be used by outpatient clinics in conjunction with a complete geriatric assessment. The items are separated into the clusters of personal hygiene, health habits, and social functioning, and a lower test score indicates a higher possibility of self-neglect, with the cut-off threshold to indicate that self-neglect is present at or below 7 (77). Given the nature of outpatient clinics, a screening tool with 11 items is ideal to aid in identification of individuals who need a referral for more in depth assessments and care. However, there are limitations noted by these authors as IMSelf-Neglect relates to this conceptual framework including a lack of comprehensiveness and a lack of classification of scores on the self-neglect continuum except for the cut-off threshold at 7 indicating that self-neglect is present.

The Chicago Health and Aging Self-Neglect Instrument is a 15-item assessment that encompass personal hygiene, health habits, behavioral characteristics, environmental characteristics, and financial independence. Each item receives a rating of 0–3 to notate no risk, mild risk, moderate risk, or severe risk to health and safety without additional assistance for a score range of 0–45 (6). The data could also be classified with scores of 1–15 indicating mild self-neglect, 16–30 indicating moderate self-neglect, and 31–45 indicating severe self-neglect (50). This assessment tool quantitatively classifies self-neglect severity as mild, moderate, or severe.

Each of these assessment tools has strengths, and many aspects of the tools overlap with one another while retaining their own distinct elements. With the exception of the IMSelf-Neglect questionnaire, which was developed for a specific niche, all the tools were meant to be generalizable. There is no clear “gold standard” tool to assess self-neglect, so the variety of options, while beneficial for researchers and healthcare providers who may wish to tailor an assessment to best fit the individual or patient who is self-neglecting, does nothing to provide a solution to the quandary of standardizing the self-neglect assessment. For the purposes of identifying severe self-neglect, however, the Chicago Health and Aging Self-Neglect Instrument is the only assessment tool which provides a numerically-based system to differentiate severe from mild or moderate self-neglect.

Besides the aforementioned study by Lee et al. (72), these authors found only one other study on self-neglect interventions over the past decade. The evaluation of the elder abuse intervention program ECARE, Eliciting Change in At-Risk Elders, proved that developing relationships with at-risk elders and/or their families prior to suggesting community-based interventions produces statistically significant improvements in the elder's status. Some participants required as long as 3 months to build a working alliance with outreach specialists before moving on to interventions. Once that alliance was formed, information about available community-based resources was provided that would enhance the elder's safety and promote autonomy in whatever regard best suited the elder's situation. The results found that elder abuse risk factors decreased throughout the intervention and almost three-quarters of participants made strides in their treatment goal and advanced through at least one of Prochaska and DiClemente's stages of change (78). This study is of particular interest and important when juxtaposed with the study by Dong et al. (49). This showed that individuals who self-neglect are at a significantly increased risk of later experiencing multiple additional forms of elder abuse, even after adjusting for confounding factors with a median time between self-neglect alone and the onset of elder abuse of 3.5 years in the study's population (49). The limitations for the analysis of ECARE as it applies to the conceptual framework of severe self-neglect are that the analysis was targeted to all forms of elder abuse, which includes but is not specific to self-neglect, and it did not account for different severities of self-neglect except as a possible causative factor in the length of time required to develop a working alliance. Further studies are needed to determine the impact of ECARE if it is focused on individual aspects of elder abuse, including self-neglect or severe self-neglect.

### Automated Monitoring Systems and Machine Learning

The interventions we have referred to in this study require the presence of clinical personnel or verification from a designated advocate. Prior to the COVID-19 pandemic, in-home care and visitation of aging patients, at least sporadically or upon government request was a common reality of Western society. However, the pandemic exacerbated the need for interventions that do not require person-to-person contact.

In-home monitoring systems that incorporate artificial intelligence (AI), machine learning and data science became very useful during the pandemic, but new models are needed more than ever to provide better predictions of health conditions in a larger cohort of individuals (79, 80). It is well-documented that facial recognition and systems-based algorithms used in AI do not result in accurate identification or equal outcomes for black people (81–83).

## Self Neglect and the Healthcare System

A study published shortly before the onset of the pandemic showed that there was racial bias in a widely used algorithm of U.S. healthcare systems, further exacerbating racial and ethnic disparities in an affected health care population of ~200 Million people (84). Reflective of health inequities, disparities and racial bias in the U.S. healthcare system, the death rate of African-Americans, Hispanic, and Native-Americans from COVID-19 is almost 3 times that of whites (85). As of January 5, 2021, Black and Indigenous Americans experienced the highest rates of death over the past 4 weeks from COVID-19, exceeding 1 in 750 nationally (86). The need for in-home health monitoring systems that accurately reflect disease symptoms and conditions based on facial attributes and whole population analysis is immense (87).

There are reported cases of elderly abuse at nursing homes which has been poorly studied. A look at the SCOPUS database reveals that only 78 out of 1,342 published articles in the last 5 years deal with abuse of the elderly in relation to nursing homes, representing a very small part (5.81% of the considered sample as compared to the number of articles on child abuse highlighting the lack of interest of research on this phenomena) (88).

A 41- Question survey was administered to healthcare professionals, working at the Internal Medicine and Geriatric Wards of two different university Hospitals of Southern Italy, representative of the Italian health public system. For the majority healthcare workers, neglect represented a type of abuse, whereas 40% of physicians and 37% of nurses considered this concept false. All professionals recognized the elder abuse as violation of basic human rights, but 46.94% were not sure about the existence of standard procedure for abuse reporting and treatment. The healthcare workers did not take any necessary action, neither report them to public authorities nor adult protective service agencies. There is still a strong need for education and specific training programs on elder abuse (89).

### Parsimony

Considering the complex nature of severe self-neglect, this framework is not concise. If researchers are interested in mild self-neglect, one assumes a more concise model may be developed. What shocks communities and health care professionals is the extreme form of self-neglect since no prior research has been conducted on its etiology. A few published case studies exist on treatment modalities (70, 71, 90).

### Testability

This framework has great potential for future testing. It expands on the previously published CREST model of self-neglect to include a definition, predictors, and the refusal component as well as the manifestations. Each domain can be tested individually, in parts, or as a whole. For example, elders who develop ED can be followed to determine if self-neglect ensues, or self-neglecters can be tested to determine if they have ED. Further, prospective longitudinal studies can be conducted to determine what risk factors or circumstances initiate the onset of self-neglect. Can early intervention prevent or abate this problem?

### Usefulness

Utilization by medical, social, nursing, and legal groups may benefit from this framework by having a better understanding of what occurs before the manifestations of severe self-neglect. This framework can be used to teach community agencies about the critical manifestations of which workers must be aware in order to identify severe self-neglect. For example, postal workers have an excellent opportunity to peer into an elder's home during routine mail or package delivery. Emergency medical personnel and home health care workers are in a particularly unique position to be able to assess the physical manifestations of self-neglect in the home from a medical perspective if their services are required by the individual. The primary goal of this framework is to illustrate that this disorder is not a life-style choice.

### Generalizability

Currently, this framework is specific to severe self-neglect. It cannot be applied to other forms of elder abuse such as financial exploitation or physical abuse. It shares some features of elders experiencing caregiver neglect such as malnourishment, delirium, and untreated medical conditions. Extreme caution is warranted if applying this framework to mild or moderate self-neglect as signs and symptoms may differ or be much less apparent. Future research should focus on adapting this framework to abuse by caregivers. Additionally, future studies should be conducted regarding the reversal of self-neglect and determining if there is a point of severity on the self-neglect continuum at which abatement is no longer a viable option to provide a point of reference for healthcare providers and social workers in determining how best to treat or assist in cases of self-neglect.

### Conclusion

A conceptual framework and a new definition for severe self-neglect is proposed as a foundation for further research. This framework can be used in clinical and community settings to aid health care professionals in identifying severe self-neglect. In doing so, treating these individuals may prevent an early death and possibly reverse this complex health problem. Current knowledge of severe self-neglect is based on studies of individuals already in a state of severe neglect. Collaboration among different disciplines is needed to intervene in these complex cases in order to arrest or reverse the progression of self-neglect. The nursing, medical, and social literature is lacking in prospective, longitudinal data, which must be addressed in the near future.

## Data Availability Statement

The original contributions presented in the study are included in the article/supplementary material, further inquiries can be directed to the corresponding author/s.

## Author Contributions

SP provided substantial contributions to the conception of the work, acquisition and analysis of data for the work, and is the corresponding author on this paper who agrees to be accountable for all aspects of the work in ensuring that questions related to the accuracy or integrity of any part of the work are appropriately investigated and resolved. MD provided substantial contributions in the acquisition and analysis of data for the work. EJ and FJ provided substantial contributions to the design of the work, interpretation of the data for the work, and revised it critically for intellectual content. FJ also serves as a corresponding author on this paper who agrees to be accountable for all aspects of the work in ensuring that questions related to the accuracy or integrity of any part of the work are appropriately investigated and resolved. All authors contributed to the article and approved the submitted version.

## Conflict of Interest

The authors declare that the research was conducted in the absence of any commercial or financial relationships that could be construed as a potential conflict of interest.
